# Medical News and Bulletin

**Published:** 1843-11-25

**Authors:** 


### MEDICAL NEWS AND BULLETIN.

THE CLINICAL LECTURES.

We regret that we have been obliged to disappoint our readers in the present number, owing to an unavoidable mistake with regard to the Clinical Lectures. We have made arrangements by which this cannot possibly happen for the future. In our next number we shall publish two if not three lectures. The lectures will be, throughout, revised and corrected by the lecturers themselves, expressly for this Journal.

LOUISVILLE MEDICAL INSTITUTE.

Students are coming in well to this Institution, and the class promises to be one of the largest that has yet attended it.

AFFECTATION OF WOULD-BE "SAVANS."

A short time ago there was a long and unmeaning article by M. *Guerin*, in the Gazette Medicale, of which he is the "redacteur en chef," with this extraordinary heading—"of the scientific unity and solidarity (!) of anatomy, physiology, pathology, and therapeutics in the study of the phenomena of the animal organism." We have rarely met with such a tissue of pompous absurdity—it possesses the rare union of German unintelligibility and French verbosity. As a matter of course, the "refrain" of the whole is something about muscular retraction, deformities, and subcutaneous section of muscles, &c. A brick will suffice as a sample of the building: "The anatomy of ages has shewn me that, from early fœtal to adult life, the fibrosity of muscles in all animals alike goes on incessantly increasing, in relation to the fleshy constitution, that is to say in the ratio of the antiquity and the intensity of action of the cause." Reader, can'st thou tell us poor mortals what lingo this is; for we frankly confess that we do not understand one word of it? Surely this is the "darkening counsel by words without knowledge," that the wise man of old hath so well exposed.—*Medico-Chirurgical Review.*

DROLL REQUEST OF A LEARNED ACADEMICIAN.

"M. *Baudelocque* requests of the Academy that it will appoint a committee, before which he undertakes to prove that he never loses either a woman or an infant after delivery, whether this has been natural, or complicated with hæmorrhage, convulsions, præternatural position of the child, &c., *provided* he has been called in from the very commencement of the accident, and before any attempt has been made to terminate the accouchement. As a matter of course, he excepts from his proposal all cases of deformity of the pelvis, and of extra-uterine pregnancy."

Well, this modest request out-herods all the regular professional puffs we have ever met with. Hide your diminished heads, all ye Hygiests, Homœpathists, Hydropathists, &c. &c. for most of you admit that you do meet with occasional failures; but here is a hospital physician tells us that he never does! These Frenchmen are surely strange mortals.

*Ibid.*

M. BUFFALINI, OF FLORENCE.

One of the most eminent physicians of Italy in the present day is M. *Buffalini* of Florence. His clinical lectures draw a large concourse of students, and his fame nearly equals that of his distinguished rival of Parma, *Tommasini.* He is generally regarded as "un medicin organicien;" and yet his lectures and practice savour strongly of the Essentialist school. In commenting upon any case of fever, he pays minute attention to every appreciable alteration alike of the fluids and of the solids, and he does not hesitate to acknowledge openly that the changes observable, either in one or the other, are by no means uniformly in accordance with the severity of the constitutional affection, or can be considered as the proper *causes* of it. In many respects he is a decided Humoralist; but, while leaning strongly to the opinion that most fevers are attributable to certain changes in the circulating fluid, he on the whole does not trouble himself much with prying into the nature of morbid individualities, but contents himself with scrutinising their exciting causes, the various symptoms which they exhibit, and with devising the means best fitted for their relief. Every one, who has had an opportunity of witnessing his practice, speaks in high terms of it; avoiding the pernicious errors of Contra-stimulism, (which he has contributed in no small degree to throw into discredit,) he shews great discretion and tact in the use of his remedies. He has published an able work entitled "*Des fondemens de la Pathologie Analytique.*"—*Ibid.*

THE MEDICAL PRESS IN ITALY.

Italy possesses more journals of medicine and pharmacy than France does; (we certainly were not prepared for this announcement;) but the division of the country into so many separate states places a great bar to their diffusion and general usefulness. The medical press suffers much from the consequences of the general political organism of the country; it has not a few obstacles to overcome, and a good many dangers to avoid. Thus, for example, when Homœopathy first made its appearance at Milan, under the patronage of the Austrian government, the subject, it was soon found, could not be discussed in all its bearings with perfect freedom. The whole business of journalism is fettered and cramped by restrictions and vexatious annoyances. Hence it is that scarcely any of the periodicals bear that stamp of originality and freedom of opinion, which constitute the main utility of such publications; and that their chief contents are mere compilations from the French, English, and German journals. As long as such a state of things lasts, we cannot look for that energy and devotedness in the cause of professional advancement which we find in the writers of France, Germany, and Great Britain. Surely medical science may fairly be regarded as a free and open field for unlimited boldness of speech and enquiry; but, alas! it has always been found that tyranny is never satisfied until it has brought everything under its iron yoke.

*Ibid,*

